# Recombination is a key driver of genomic and phenotypic diversity in a *Pseudomonas aeruginosa* population during cystic fibrosis infection

**DOI:** 10.1038/srep07649

**Published:** 2015-01-12

**Authors:** Sophie E. Darch, Alan McNally, Freya Harrison, Jukka Corander, Helen L. Barr, Konrad Paszkiewicz, Stephen Holden, Andrew Fogarty, Shanika A. Crusz, Stephen P. Diggle

**Affiliations:** 1 School of Life Sciences, University of Nottingham, Nottingham, NG7 2RD, U.K.; 2 Pathogen Research Group, Nottingham Trent University, Nottingham, U.K.; 3 Department of Mathematics and Statistics, University of Helsinki, Finland; 4 Division of Respiratory Medicine, Nottingham City Hospital, Nottingham, NG5 1PB, U.K.; 5 College of Life and Environmental Sciences, University of Exeter, Exeter; 6 Department of Clinical Microbiology, Nottingham University NHS Trust, U.K.; 7 Division of Epidemiology & Public Health, Nottingham City Hospital, Nottingham, NG5 1PB, U.K

## Abstract

The Cystic Fibrosis (CF) lung harbors a complex, polymicrobial ecosystem, in which *Pseudomonas aeruginosa* is capable of sustaining chronic infections, which are highly resistant to multiple antibiotics. Here, we investigate the phenotypic and genotypic diversity of 44 morphologically identical *P. aeruginosa* isolates taken from a single CF patient sputum sample. Comprehensive phenotypic analysis of isolates revealed large variances and trade-offs in growth, virulence factors and quorum sensing (QS) signals. Whole genome analysis of 22 isolates revealed high levels of intra-isolate diversity ranging from 5 to 64 SNPs and that recombination and not spontaneous mutation was the dominant driver of diversity in this population. Furthermore, phenotypic differences between isolates were not linked to mutations in known genes but were statistically associated with distinct recombination events. We also assessed antibiotic susceptibility of all isolates. Resistance to antibiotics significantly increased when multiple isolates were mixed together. Our results highlight the significant role of recombination in generating phenotypic and genetic diversification during *in vivo* chronic CF infection. We also discuss (i) how these findings could influence how patient-to-patient transmission studies are performed using whole genome sequencing, and (ii) the need to refine antibiotic susceptibility testing in sputum samples taken from patients with CF.

The Cystic Fibrosis (CF) lung presents a complex polymicrobial ecology, which in turn complicates the treatment of chronic bacterial respiratory infection. The pathogen most commonly associated with CF is *Pseudomonas aeruginosa*. Ubiquitous in the environment, *P. aeruginosa* is one of the leading causes of opportunistic infections in humans^1^, and is a major cause of increased morbidity and mortality in patients with CF^2^. Its ability to colonise pulmonary epithelial cells, communicate using quorum sensing (QS) signals and form multicellular biofilms, plus its innate resistance to many antimicrobials, results in chronic infections that are almost impossible to eradicate, leading to a clinical pattern of intermittent exacerbations and an eventual decline in lung function^2^,^3^,^4^. Furthermore, initial colonising strains of *P. aeruginosa*, thought to be acquired from the environment, have the potential to be displaced by more virulent and transmissible epidemic strains^5^,^6^,^7^.

It is now well established that founder *P. aeruginosa* populations evolve over many years of chronic CF infection, leading to high levels of temporal phenotypic and genetic diversity within a single patient^5^,^8^,^9^,^10^,^11^,^12^,^13^,^14^,^15^. Longitudinal genomic studies of single colonies isolated from individual CF patients suggest such variation is derived from a mixture of single base pair mutations, insertion and deletion events and recombination events^10^,^16^. Additionally, recent studies have examined the diversity of *P. aeruginosa* populations isolated from individual patients at a single time point^14^. These suggest that considerable phenotypic variation exists at any given time and Pulsed-Field Gel Electrophoresis (PFGE) is suggestive of some level of genetic variation^14^. Despite these studies, it is still not clear how such diversity arises and how this impacts on clinically important factors such as identifying transmission events between patients and performing reliable antibiotic susceptibility testing.

Recent studies examining diversity within *P. aeruginosa* populations isolated from the CF lung have described significant variation in antibiotic susceptibility profiles in isolates which vary in morphological appearance. The most commonly described difference is that observed between mucoid and non-mucoid colonies^2^,^9^,^17^. However, no study has conducted a detailed examination of a single, morphologically homogeneous population of *P. aeruginosa*, nor has any study provided a comprehensive map at the genome level of phenotypic variation within an extant CF lung population.

Here we address this gap in our understanding of how diversity evolves in the CF lung and how this may impact on decisions made in a clinical setting, where microbiological analyses are often based on a single colony. We have comprehensively analyzed the levels of phenotypic and genotypic diversity within a single CF lung ecosystem at a single snapshot in time. We reveal previously unobserved correlations and trade-offs between the production of different virulence-related exoproducts, quorum sensing (QS) signals and resistances to a range of clinically-relevant antibiotics. Furthermore, we show significant increases in antibiotic resistance when multiple isolates are cultured together. Finally, we use a range of genomic analyses to quantify genotypic diversity within this single patient. These reveal (i) the dominant role of recombination over spontaneous mutation in the generation of *in vivo* diversity; (ii) unexpected and complex relationships between genotype and phenotype; (iii) the difficulty of classifying patient-to-patient transmission events based on the analysis of single colonies; and (iv) the likely underestimation of antibiotic resistance based on testing a single or few colonies per patient.

## Results

### *P. aeruginosa* isolates from a single sputum sample display phenotypic tradeoffs

**Figure 1:**
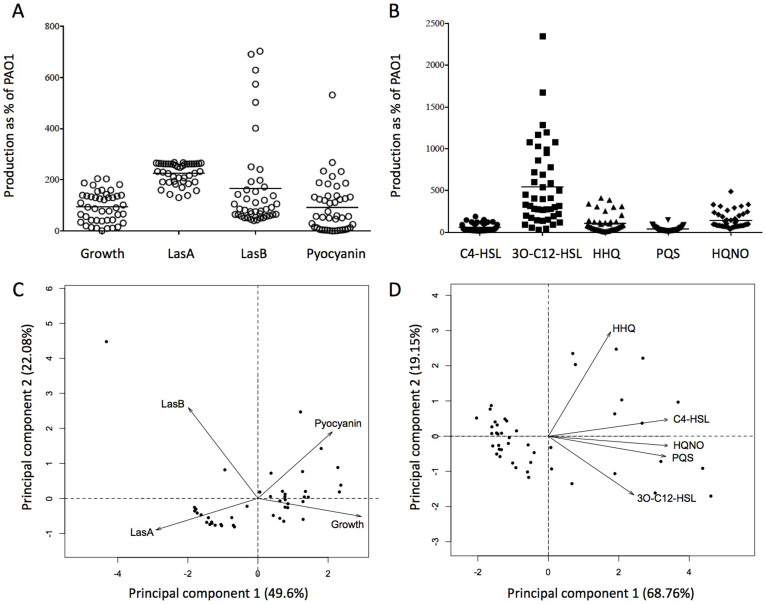
Phenotypic diversity of *P. aeruginosa* populations. We measured growth, exoproducts and QS signal molecules of individual isolates and expressed these as a percentage of the values obtained for a PAO1 wildtype. Each data point represents the mean of triplicate assays for an individual isolate. (A) Phenotypic assays for growth and exoproduct production show that isolates display large variation between individuals when compared with the PAO1 reference strain. (B) QS Signal molecule measurements show that variation occurs between isolates when compared to the PAO1 reference strain. Panels (C) and (D) show the results of PCA on phenotypic and QS signal data respectively. PCA reduces multiple variables (four phenotypic traits or five QS molecules) to two dimensions, allowing us to plot multivariate data on simple *x,y* coordinates. The arrows are vectors that show how the original variables relate to the new *x* and *y* axes. The PCA plots show that QS signals are linked and that tradeoffs exist between other phenotypes measured.

We randomly selected 44 morphologically identical, non-mucoid *P. aeruginosa* colonies (hereafter referred to as isolates) from a single spontaneously expectorated sputum sample from a clinically stable CF patient with a chronic *P. aeruginosa* infection. We assayed each isolate for overnight growth in standard laboratory medium and for a selection of phenotypes that have previously been associated with virulence in CF infection. The isolates displayed considerable variation in growth and also in the production of tissue-degrading proteases (LasA protease and LasB elastase), the redox-active toxin pyocyanin and the QS signal molecules *N*-butyryl-L-homoserine lactone (C4-HSL), *N*-(3-oxododecanoyl)-L-homoserine lactone (3O-C12-HSL), 2-heptyl-3-hydroxy-4(1*H*)-quinolone (PQS), 2-heptyl-4-hydroxyquinoline *N*-oxide (HQNO) and 2-heptyl-4-quinolone (HHQ). These data are summarised in Fig. 1a and b.

We then wished to determine the extent to which the expression of different virulence factors covaried. On the one hand, the variation shown in Fig. 1a and b could reflect a set of isolates with phenotypes that range from generally low virulence (poor growth and low levels of virulence factor expression) to generally high virulence (extensive growth and high levels of virulence factor expression). On the other hand, the isolates could have qualitatively different phenotypes – e.g. some may produce lots of protease but very little pyocyanin, while others may show the opposite pattern, suggesting that expression of one phenotype could be traded off against another. To address this question, we conducted principal component analyses (PCA).

The results of a PCA on growth and three virulence-associated exoproducts (LasA, LasB and pyocyanin) are shown in Fig. 1c. This revealed negative correlations between the traits, such that no individual isolate demonstrated high values for all four variables. In particular, higher growth was associated with lower per-cell production of LasA protease and LasB elastase, whilst higher pyocyanin production entailed lower LasA protease production: these relationships are illustrated by the vectors for the original variables pointing away from each other on the PCA plot. The first two principal components explained approximately 70% of the total variation in these phenotypes. Pairwise Spearman's rank correlations produced results consistent with the PCA and the results for phenotype-related traits were unchanged by the exclusion of one outlier (SED23) (Supplementary Fig. S1).

We conducted a separate PCA on the production of five key QS signals, which between them regulate the expression of a range of virulence factors. QS signals have previously been extracted from the sputum of CF patients^18^,^19^. Production levels of the five molecules were generally positively correlated, reflected by the close alignment of the vectors representing the original variables on the PCA plot (Fig. 1d). The first two principal components account for almost 90% of the total variation in the data set and the majority of the data points varied mainly in their level of 3O-C12-HSL production. Consistent with the PCA results, pairwise Spearman's rank correlation coefficients (Supplementary Fig. S1) revealed an almost perfect correlation between HQNO and PQS production (*r_s_* = 0.96, *p* < 0.001), both of which were also strongly correlated with the production of C4-HSL (*r_s_* = 0.89 and 0.85 respectively, *p* < 0.001) (Supplementary Fig. S1).

### Whole genome SNP typing shows high levels of diversity and signatures of adaptive events in the extant *P. aeruginosa* population

**Figure 2:**
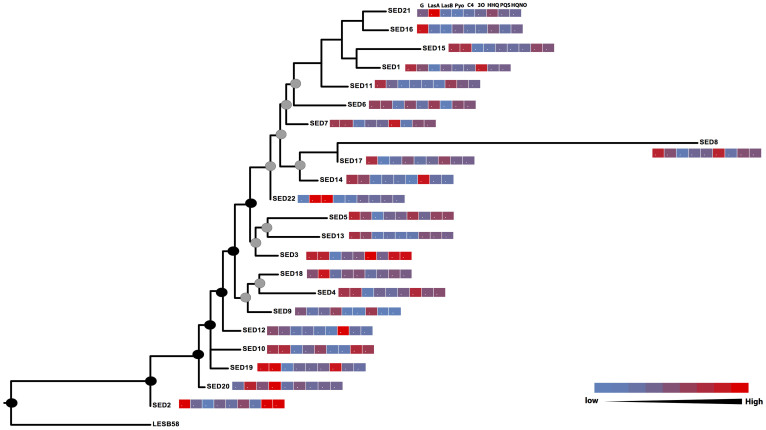
Maximum likelihood phylogeny based on SNP typing of the 22 sequenced isolates against LESB58. Nodes obtaining >95% bootstrap support (black circles) and >75% bootstrap support (grey) are indicated on the tree. The heatmaps accompanying each taxa represent the levels of production of each phenotype relative to the reference PAO1 strain and are indicated above SED21. Abbreviations: G (growth), LasA (LasA protease), LasB (protease), Pyo (pyocyanin), C4 (C4-HSL), 3O (3O-C12-HSL).

To contextualize the high levels of phenotypic diversity observed within this single sputum sample, we performed whole genome sequencing on 22 of the 44 isolates (SED 1–22), which were selected to represent the full spectrum of phenotypic diversity. Both methods confirmed that all isolates were close relatives of the Liverpool Epidemic Strain (LESB58), a particularly aggressive epidemic clone of *P. aeruginosa*^20^. As such, we performed mapping of raw sequence data for all 22 variants against the LESB58 reference genome using SNP calling parameters equivalent to those used in recent genomic epidemiological studies of microbial infection^21^,^22^. The resulting phylogeny (Fig. 2) and SNP distance matrix (Supplementary Fig. S2) confirmed that the patient was initially infected with a clone very similar to LESB58, which then most probably diversified within the lung, resulting in considerable levels of intra-population diversity. There were a total of 121 high-resolution SNPs called across the population, with 24 of those present across all isolates compared to the reference and so considered ancestral variants. None of the 22 isolates had a SNP difference range within 5 SNPs of the LESB58 reference strain and the most extreme differed by 64 SNPs (Supplementary Fig. S2 and Supplementary Table S1). Of the 77 SNPs which varied across the isolates, 45 were present in multiple isolates randomly distributed across the phylogeny (Table 1).

Despite all of these variants originating from the same single founder clone, such dissemination of SNPs is reminiscent of homoplasic mutation, where mutations in the same nucleotide occur independently in phylogenetically distinct individuals. A number of the homoplasic-like sites also appeared to be under the force of positive selection as determined by dN/dS ratios. This indicated that non-synonymous mutations in *mexB*, PLES_11151, PLES_28341, *glyA2*, and PLES_59241 all displayed signatures of adaptive evolution in this extant population with dN/dS ratios far in excess of 1. Attempts to superimpose the observed phenotypic diversity of the 22 isolates onto the phylogeny failed to show any pattern of acquisition or accrual of phenotypes along the evolutionary trajectory of the phylogeny, with phenotypes appearing randomly across the tree (Fig. 2). This is consistent with our observations of homoplasic-like accrual of mutations which can occur as a result either of fixation of selectively advantageous mutations, or by intra-population recombination. None of the high stringency SNPs identified in the mapping process showed a meaningful biological correlation with the phenotypic diversity. The vast majority of isolate-unique SNPs occurred in SED8, however this isolate showed no mutations associated with hypermutation (nor did any other isolate) and all isolates failed to display classical hypermutation phenotypes when analysed using standard rifampicin spontaneous resistance testing (data not shown).

### Analysis of *de novo* assembled genomes indicates a dominant role for recombination in generating diversity in the extant population

**Figure 3:**
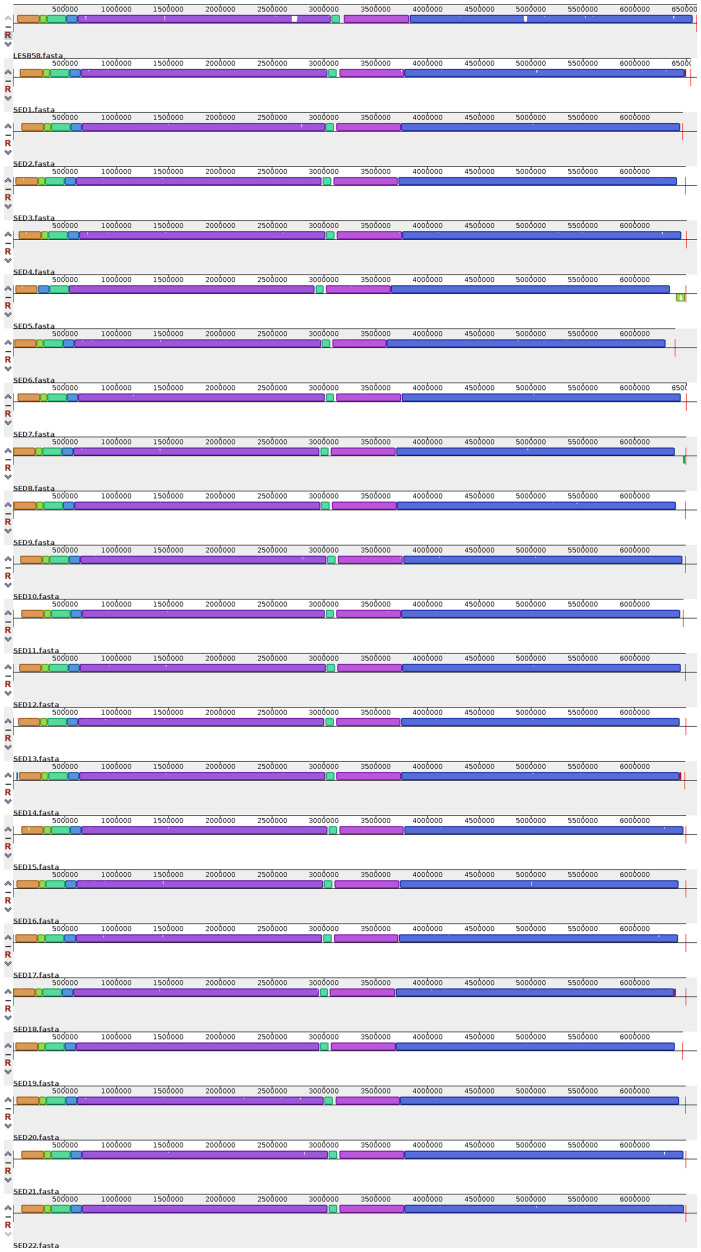
Whole genome alignments of the de novo assembled genomes of 22 isolates and the reference genome LESB58. Alignment was constructed using Progressive Mauve, and local collinear blocks containing orthologous sequence are colour coded.

Whilst high resolution SNP typing gives the most accurate determination of variation within the population, it does not take into account large-scale insertion, deletion, and chromosomal rearrangement events which may generate diversity. Corrected *de novo* genome assemblies were constructed for each isolate using Velvet and PAGIT, and from these, whole genome alignments were performed using progressiveMauve and pairwise Blast comparisons using Blast Ring Image Generator (BRIG). Both comparative methods showed a lack of large-scale insertion or deletion events responsible for the phenotypic diversity. The loss of a 26.4 Kbp cryptic phage in isolates SED2, SED11 and SED19 compared to LESB58 is the most obvious region of difference (Supplemental Fig. S3). However this event shows no correlation to any phenotypic pattern seen in these isolates. Perhaps more significantly, there are no acquisition events in any of the 22 variants sequenced as determined by a Progressive Mauve alignment of the de novo assembled sequences (Fig. 3). Given that large-scale acquisitions and deletions are well documented in longitudinal CF *P. aeruginosa* populations, we further confirmed our finding by constructing a pan-genome of the de novo assembled genomes, our 22 variants and LESB58, and plotted the presence and absence of every locus as a heat map (Supplemental Fig. S4). Our data clearly shows there are no large genomic islands that have been acquired by any of the variants. There are a small number of genetic loci which appear to be strain specific, however BLAST analysis identified these as genes with highly divergent sequences compared to the ortholog in LESB58.

We also checked for major chromosomal inversions and rearrangements which may confer intra-population diversity by utilising our paired-end sequencing reads and performing break-point analysis on each de novo assembly compared to the reference LESB58 genome (Supplemental Fig. S5). Our analysis suggests the presence of just a single observable inversion event in isolate SED5 with the genomic architecture across all 22 variants almost identical by our analysis. Finally we checked for subtle differences, which may result in frameshift mutations. Our previous SNP analysis identified just one mutation in the *mexB* gene present in 2 variants, which introduced a premature stop codon, and two SNPs affecting intergenic regions possibly affecting gene expression. To confirm there were no small indel events causing frame shifts, we systematically compared each of the 22 variant genomes against LESB58 using Artemis Comparison Tool (ACT) looking for truncated ORFs or CDS mergers, as well as indels in intergenic regions in the variants, however no such frameshift mutations were found in the data set.

Given the identification of a number of regions with highly divergent sequence in the variants compared to LESB58, and the earlier identification of homoplasic-like mutations randomly distributed across the population phylogeny, we determined the levels of recombination in the de novo assembled genomes. BRATNextGen analysis on a core genome alignment detected recombination events in all genomes ranging from a single event in SED19 to seven recombination segments in SED18 (Supplemental Fig. S6), with almost all detected recombinant segments shared across some members of the data set, indicative of intra-population recombination. From the alignment of corrected genome assemblies, there were a total of 1436 single nucleotide polymorphisms (SNPs) across the 22 genomes, with 1296 present in regions identified as recombining, giving an r/m value of 9.2571. This indicates that the SED isolates are approximately ten times more likely to have acquired genetic variation by recombination than by spontaneous mutation. More importantly of the 45 high resolution SNPs exhibiting homoplasic-like properties, 40 were located on recombining regions, supporting the likelihood that mutations are being propagated across the extant population by recombination.

### Phenotypic diversity in the extant *P. aeruginosa* population does not associate with previously reported and associated mutations

**Figure 4:**
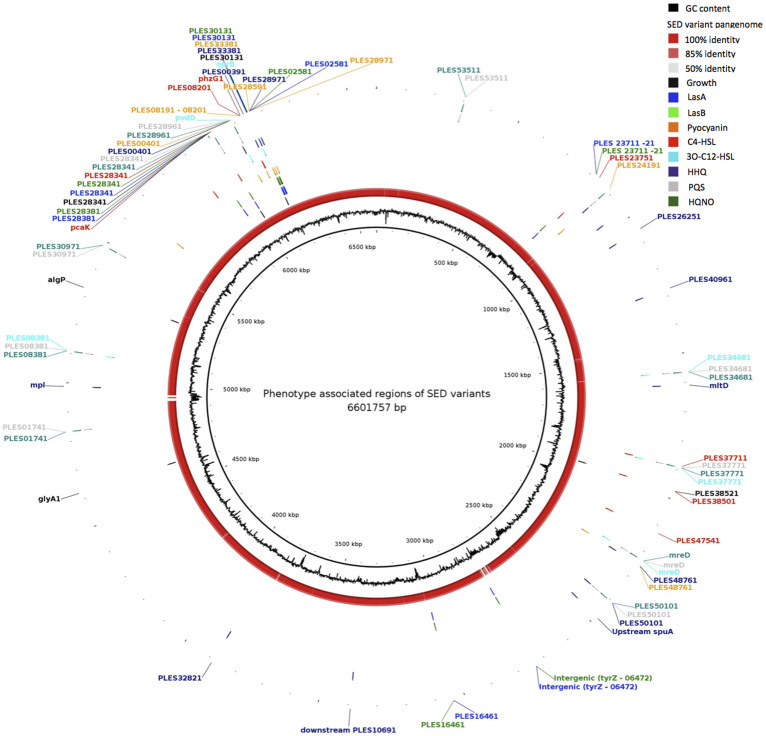
Circular representation of regions identified as undergoing recombination relative to the LESB58 reference genome. Regions statistically associated with phenotypes are indicated in the concentric ring representing that phenotype (Growth innermost to HQNO outermost). The ORFs encoded in recombining regions are indicated by their gene name as annotated in LESB58. The Gene name-tags are colour coded to indicate clearly the phenotypes they are associated with.

As mentioned previously, attempts to superimpose the observed phenotypic diversity of the 22 isolates onto the phylogeny failed to show any pattern of acquisition or accrual of phenotypes with variable phenotypes appearing randomly across the tree (Fig. 2), which is consistent with dissemination via recombination. None of the high stringency SNPs identified in the mapping process showed a correlation with the phenotypic diversity we observed as reported in classical bacterial genetics experiments. Given our observation of significant intra-population recombination, we investigated if there was a statistical correlation between recombination events and phenotypic diversity. We utilised the widely accepted technique of performing permutation tests of phenotypes against the whole genome alignments^23^ and identified between 5 (LasA production) and 15 (HHQ production) recombining regions significantly associated (p < 0.05) with variation observed in a given phenotype (Fig. 4; Supplementary Table S2), including regions enriched for homoplasic mutations in the high-confidence SNP typing data (Table 1).

Each of these recombining regions was mapped onto the LESB58 reference genome, allowing us to identify the CDS present in each region (Fig. 4). This analysis showed a high degree of overlap in the recombining regions associated with different phenotypic changes. Of particular interest are the recombining loci significantly associated with altered levels of production of PQS and HQNO which are completely identical (Supplementary Table S2), suggesting these two phenotypes are intrinsically linked. This observation matches our phenotypic observation that PQS and HQNO production are almost perfectly correlated (Fig. 1d and Supplementary Fig. S1). Though a small number of recombining loci are associated with a change in just one phenotype, a total of 19 recombining CDS are significantly associated with an alteration in 2 or more observed phenotypes. Of equal importance is that none of the loci have been associated with such phenotypic properties by way of classical bacterial genetics and mutagenesis studies.

### Phenotypic and genetic diversity results in variation in antibiotic susceptibility between isolates

**Figure 5:**
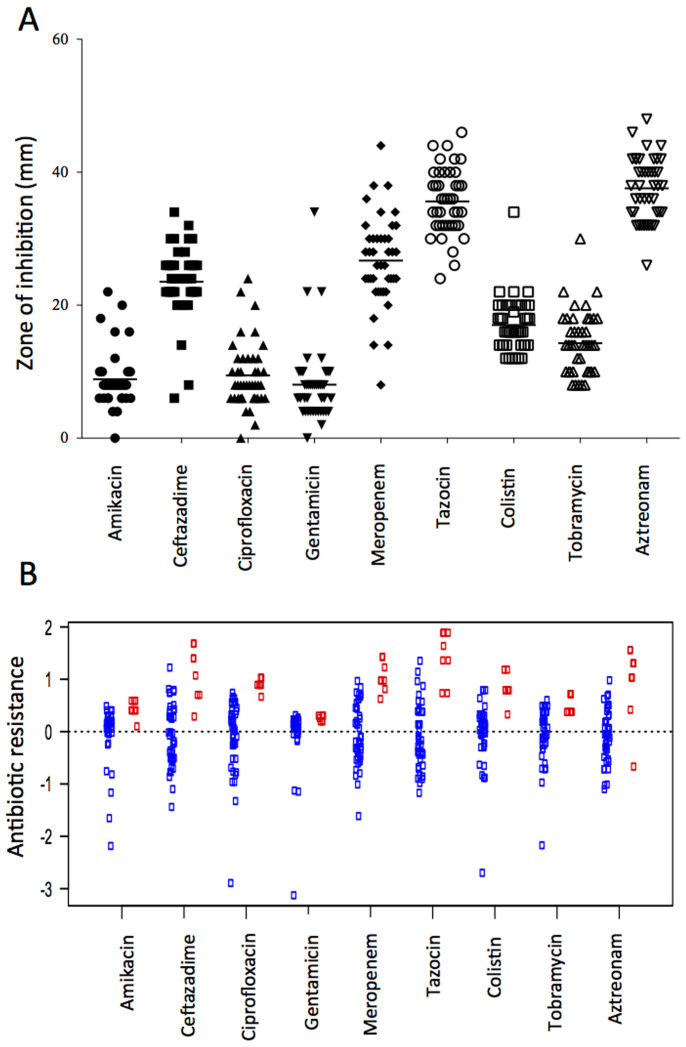
Antibiotic resistance profiles for 44 isolates measured using the BSAC method. (A) The antibiotic susceptibility of single isolates to common CF therapeutics. The recorded zones of inhibition and mean value for each 44 individual isolates are shown. Each data point represents the mean of three independent biological replicates for each individual isolate. (B) Resistance profiles of each individual isolate to 9 antibiotics compared to a mixed community of all 44 isolates. Each blue point represents the sensitivity profile of a single isolate and red points represent 7 independent measurements of a mixed community containing all 44 isolates. In both (A) and (B), the values for individual clones represent the mean of three independent biological replicates. Individual clones consistently underestimate the resistance of the mixed community.

Previous studies have shown that there is little correlation between antibiotic sensitivity patterns of *P. aeruginosa* isolated during a pulmonary exacerbation and the subsequent clinical response of the patient^24^,^25^ and reproducibility of the diagnostic test itself has been shown to be low between sample replicates^26^,^27^. We determined the resistance of our 44 isolates to 9 commonly used CF therapeutic agents using the BSAC disk diffusion method. We noted large variances in susceptibility between isolates according to the zone of inhibition produced (Fig. 5a). Pairwise correlation analysis revealed several significant positive correlations indicating the tendency for some isolates to have relatively high resistance to multiple antibiotics whilst others had relatively low resistance to multiple antibiotics (Supplementary Fig. S7). Correlations in resistance were also found between antibiotics with similar mechanisms of action (such as the aminoglycosides).

We then mixed all 44 isolates as a population for comparison with individual isolates and repeated the antibiotic susceptibility testing. We found that variation in the zone of inhibition was reduced in mixed versus single isolates, indicating that picking colonies at random and mixing them together as a population gives a more consistent antibiotic resistant phenotype (Fig. 5b and Supplementary Fig. S8). Furthermore, we found that mixing all 44 isolates together resulted in a level of antibiotic resistance that was higher than the mean of the individual isolates tested (t_17,450_ = −2.208, p = 0.028), indicating that testing at the individual isolate level may underestimate resistance (Fig. 5b). The complexity of the antibiotic resistance variation observed across all our isolates meant that any attempts to significantly associate recombination events to specific changes in resistance patterns proved unsuccessful. This is almost certainly due to the restricted size of our data set, with recent GWAS studies mapping specific recombination and mutation events to changes in susceptibility profiles requiring thousands of isolates to achieve statistical significance^28^,^29^.

## Discussion

A number of recent studies have shown extensive phenotypic variation between different *P. aeruginosa* CF strains and isolates of the same strain taken from single patients^5^,^8^,^9^,^11^,^12^,^14^,^30^,^31^. Despite all these studies, the reasons for this phenotypic diversity remain poorly understood. In addition, there is little to no information available about whole genome resolution of the extant diversity of *P. aeruginosa* within a CF lung at any given time point. Here we show that *P. aeruginosa* intra-host diversification is strongly linked to recombination. Our findings have broad implications for (i) classical phenotype/genotype correlations; (ii) informing contact transmission networks and (iii) routine antibiotic susceptibility testing.

We began our study by taking 44 morphologically identical non-mucoid ‘isolates' from a single sputum sample, obtained from a patient with CF, known to be chronically infected with *P. aeruginosa* for approximately 3 years. At the time of sampling the patient was clinically stable and was prescribed standard maintenance oral and nebulised therapies only. Although all of the isolates appeared morphologically indistinguishable on agar plates, with no small colony variants, they actually displayed extensive phenotypic variation when screened for growth, pyocyanin, LasA and LasB production and QS signal molecule production. This is in broad agreement with other recently published work. For example, it has been reported that the majority of phenotypic diversity occurs within patients rather than between patients^9^ and that identical colony morphotypes from a given patient demonstrate a large degree of phenotypic variation^14^.

We used our phenotypic data set to determine whether there were correlations between (i) phenotypes and (ii) QS signal molecules. Extensive molecular studies over the past 20 years have shown that particular traits are linked by complex genetic networks. For example, the production of different QS signals have been shown to be linked, whilst QS links key phenotypes such as LasB (elastase) and pyocyanin^32^,^33^. The majority of these studies have been undertaken using the laboratory standard *P. aeruginosa* strain PAO1, a wound isolate taken during the 1950's^34^. Using PCA we found positive correlations between five key QS signal molecules produced by *P. aeruginosa*, with particularly strong correlations between PQS and HQNO and PQS and C4-HSL. These positive correlations broadly fit with what has been described in the literature for PAO1. In contrast we found trade offs between the other phenotypes tested (growth, LasA, LasB & pyocyanin). For example, increased growth resulted in a reduced per cell production of both LasB and LasA (Fig. 1). Our results demonstrate that phenotypic correlations found in well-studied laboratory wild types of *P. aeruginosa* do not necessarily match in *P. aeruginosa* strains recently collected from *in vivo* infections. The reasons for these trade-offs are unknown, but a number of factors, such as within-host competition, lung spatial structure, host immune response, transmission events and host survival, could produce evolutionary trade-offs between phenotype and parasite virulence^35^,^36^.

We attempted to contextualize our phenotypic observations by performing genome sequencing on 22 isolates, which were chosen to represent the full spectrum of diversity observed. Examination of differences in gene content showed there were no gene acquisition or loss events across our isolate set. A small cryptic prophage had excised from three of the isolates but this does not correlate with any particular phenotypic pattern. Previous studies examining the diversity of LES strains from across the globe have shown far greater variation in gene content between strains with variation in large genomic islands^37^,^38^. In contrast a study of 55 isolates from 21 individuals showed no large scale insertions or deletions, as well as the presence of parallel patho-adaptive mutations^39^. PFGE based studies examining extant diversity in patients has shown variations in genomic architecture^40^. Similarly frameshifts and small indels have previously been reported to be key in generating phenotypic diversity within clonal *P. aeruginosa* populations^41^, but such genetic variation was not detected in our data set. Conversely a recent study, which investigated the experimental evolution of a *P. aeruginosa* population over time^42^, showed very few indels fix within a population over time, and that small indels resulting in frameshift mutations came under extremely strong purifying selection.

Our SNP phylogeny showed a total of 121 variable sites with respect to the LESB58 reference genome, 24 of which were common and so considered ancestral to the original infecting strain. This leaves a total of 97 differentiating SNPs in our set of variant strains. Such levels of diversity within a patient infected for 3 years seem high when one considers a recent genomic study of *P. aeruginosa* strain PA14, which reported an accumulation of only 15 SNPs over a period of 15 years^43^, and a study of global and temporally distributed LES strains which reported a distance range of 66–156 SNPs^37^. However our observed levels of extant diversity are not unreasonable if one considers the mutation levels calculated for *S. aureus* of 1 core genome SNP every 6 weeks^44^ which would equate to an expectation that any random isolate variant would differ from its ancestor by approximately 27 SNPs after the 3 year period in which the lung has been infected. This is within the magnitude of values obtained between any pair of isolates in our pairwise SNP distance matrix with the exception of SED8. The possibility also exists that the differences in SNP levels between our study and those conducted in PA14 reflect the differences in mutation accumulation occurring during the initial stages of adaptation compared to those occurring in an established infection where the infecting strain is well adapted to the lung environment.

The SNPs observed at 45 of the reference genome variant sites exhibited characteristics of homoplasy. A number of the homoplasic-like sites also displayed dN/dS ratios far in excess of 1, which is a strong signal for positive selection, suggesting these sites are undergoing adaptive evolution. Our data showing multiple homoplasic-like sites under neutral selection in combination with defined sites under adaptive evolution mirrors that recently shown in a comprehensive study of within patient diversity of *Burkholderia dolosa* within the CF lung^45^. The *B. dolosa* study was performed on multiple isolates accumulated from multiple patients over time and provided a definitive blueprint for pathogen evolution in the CF lung. Our study expands on this by examining extant isolates of a far more prevalent CF pathogen, *P. aeruginosa*, in a single patient, and provides further evidence for the forces underpinning the generation of diversity in a pathogen population in the CF lung. The vast majority of our identified SNPs occurred in hypothetical proteins as opposed to surface associated factors^45^ or drug resistance associated loci^44^.

Our finding that the vast majority of SNPs within our isolate data set are present in regions undergoing recombination also provides further resolution to the mechanisms by which diversity may be generated within the CF lung. It has long been known that genetic recombination occurs in *P. aeruginosa*^34^, and our data suggests that recombination is an important factor in the short-term generation of genetic diversity within *P. aeruginosa* isolates within the CF lung with an r/m rate approaching 10. Homologous recombination has been shown to play an important role in the early adaptation of *P. aeruginosa* to the human lung, principally in creating deletions leading to genome reduction^16^. That particular study focused on a collection of isolates from multiple patients spanning a 35-year period whereas our study focuses on the diversity sampled at a single time point, and therefore our data suggests for the first time, that recombination is extremely important in the continuous evolution of *P. aeruginosa* within individual patients with CF.

Instances of large scale acquisitions, indels, frame shifts, and chromosomal inversions affecting *P. aeruginosa* phenotypes undoubtedly exist over temporal and geographical scales, as do accumulation of adaptive mutations. However our data, and that from experimentally evolved populations, may suggest that such events occur as a result of enormous selection pressures exerted within fluctuating environments over time, and may be less commonplace in stable populations or in short term evolutionary events. Whilst it cannot be excluded that our observations are highly specific to the strain and/or patient environment we have studied, it must be noted that the populations examined are very different. Our extant diversity study highlights the levels of diversity present in a lung at any given time point. Temporal studies are more likely to identify dominant genotypes within a patient at each time point sampled, therefore identifying long term selective evolutionary events. This is even more likely if sequencing is performed on a sweep of colonies from a growth culture. As such it is possible our data set may identify loci which arise under short term evolutionary pressures as opposed to the longer term evolutionary forces exposed in temporal studies. Recent work examining the evolution of *Salmonella paratyphi*, suggested that the vast majority of mutations which occur in that pathogen population are transient, coming under positive selective pressure for a limited time-frame before being purged by negative selection due to the detrimental effect on metabolic fitness^46^. Our findings suggest that future studies that aim to analyse how a strain evolves within patients by repeatedly sampling patients over time, should consider taking multiple colonies or populations per patient at each time point to better understand how the evolutionary forces currently known to impact pathogen evolution relate to the levels of extant diversity at each time point and how these two interplay. This would provide even greater resolution into how pathogen populations evolve in response to the human host.

The majority of our high-quality SNPs mapped against hypothetical proteins and none of them mapped against genes which would normally be associated with the phenotypic changes observed in our isolates. For example, the phenotypes we measured (LasA, LasB and pyocyanin) have all been shown to be highly QS-dependent^32^,^33^. Furthermore, *lasR* mutants have been shown to accumulate in the CF lung^10^. Despite this, none of our SNPs mapped against any known genes thought be involved in QS. When we performed statistical correlations between recombining regions and observed phenotypes, we also found regions significantly associated with phenotypic variation in genetic loci not classically associated with such phenotypes. Our data set highlights the intricate complexity of genetic mutations and their knock on effects on microbial phenotypes. It also suggests that whilst classical bacterial genetic experiments accurately identify loci involved in colonization, persistence and virulence in culture and in model systems, the mutations that underlie phenotypic alteration and adaptation to the human environment may be far more subtle and likely involve genetic loci whose function we do not yet fully understand. Very recent work in *Salmonella* *typhimurium* has demonstrated that bi-stable expression of virulence factors within an infecting population leads to the formation of other beneficial phenotypic changes in sub-populations, in that instance resistance to antimicrobials^47^. It was also shown that bet-hedging of heterogeneous expression of virulence factors in a population can protect clonal populations against mutants which subvert labour division. This draws comparisons to our observations in that bet-hedging and labour division can arise through trait variations and subsequent complex interactions between them, exposing degrees of functional complexity of phenotypic variation previously unreported.

Our study also has implications for understanding infection transmission networks. Our SNP distance matrix shows that the patient was likely infected with a clone very similar to LESB58 and that this has diversified in the lung via recombination. This has a potential impact on how to accurately inform contact transmission networks, with many published studies using whole genome sequencing (WGS) to inform outbreak transmission events using a cut-off of <5 SNPs^48^, or in the exemplar case of MRSA, 11 SNPs^21^. Our data suggests that baseline levels of within-patient diversity for pathogens, infectious disease and transmission scenarios may need to be developed for meaningful WGS transmission studies. Otherwise multiple colonies or whole populations may need to be sequenced from every sample during transmission studies, to prevent transmission events from being misclassified.

Finally our work has implications for antibiotic testing. Susceptibility testing of bacterial isolates to antibiotics plays an important role within the diagnostic laboratory and helps to guide antibiotic choice for the treating clinician, and high levels of *P. aeruginosa* diversity within a patient has potential implications for routine antibiotic susceptibility testing. The validity of susceptibility testing has been challenged by several studies. Hogardt and colleagues demonstrated susceptibility results “close to random” when testing quinolones and tobramycin resistance in a selection of *P. aeruginosa* CF isolates^49^, whilst a study conducted in 1994 by Morlin compared individual morphotypes and mixed morphotypes, finding that when predicting resistance to a panel of antibiotics, the assay was far less accurate^50^. In the U.K., the current standard laboratory protocol for the testing of CF sputa relies on the selection of 1 or 2 colonies of different morphotypes in combination with the BSAC disk diffusion method to examine resistance to chosen CF therapeutics. Previous studies have shown that there is little correlation between antibiotic sensitivity patterns of *P. aeruginosa* isolated during a pulmonary exacerbation with the clinical response of the patient^24^,^25^ and reproducibility of the diagnostic test itself has been shown to be low between sample replicates^26^,^27^.

We have built on this previous work and shown that isolates from the same patient display considerable variation in susceptibilities across a broad range of commonly used therapeutics. Pairwise correlation analysis showed that if an individual isolate was resistant to one antibiotic, it was also likely to be resistant to another. Correlations in resistance were also found between antibiotics with similar mechanisms of action. We then mixed all 44 isolates as a population and repeated the antibiotic susceptibility testing in an effort to compare the level of antibiotic resistance between individual isolates and when mixed as a population of isolates. We found that variation in the zone of inhibition was reduced in mixed versus single isolates, indicating that picking colonies at random and mixing them gives a more consistent measure of antibiotic resistance. Furthermore, we found that mixing all 44 isolates together resulted in a level of antibiotic resistance that was higher than any individual isolate tested.

Overall, the literature now highlights the current inadequacies of performing antibiotic susceptibility tests with single isolates or morphotypes to guide clinical decisions regarding antibiotic choice in chronic *P. aeruginosa* infections in CF. It suggests that antibiotic sensitivity testing could be improved by testing multiple colonies or populations of *P. aeruginosa*, and that this may lead to better clinical outcomes in the future. Although ultimately this study provides detail about the mechanisms of *P. aeruginosa* evolution in the CF lung, our findings may also have broader implications for chronic *P. aeruginosa* infections in other clinical settings such as non-CF bronchiectasis, chronic obstructive pulmonary disease and burns patients.

## Methods

### Bacterial strains and culture conditions

PAO1 was used as a control strain for phenotypic assays. All clinical isolates were provided in collaboration with Nottingham University Hospitals NHS Trust. Spontaneous sputum samples were obtained from a 19 year old female patient with CF, chronically infected with *P. aeruginosa* for 3 years. At the time of sampling, the patient was clinical stable and was prescribed maintenance oral and nebulised therapies only. Sputum samples were treated with the mucolytic agent Sputasol®. Equal volumes of Sputasol (Oxoid) to sputum were added and incubated at 37°C, 250 rpm for 20–25 minutes until microcolonies were suitably broken down. Serial dilutions in 900 μl of Phosphate Buffered Saline (PBS) (Oxoid) were made from a starting concentration of 900 μl PBS and 100 μl microcolony sample 10^−1^ through to 10^−10^. For each dilution, 100 μl was plated onto selective *Pseudomonas* Isolation Agar (PIA) and spread evenly using a sterile plastic spreader. One duplicate of each concentration per isolate was performed. For growth, pyocyanin, LasA and LasB assays and QS signal molecule extractions, pre-cultures grown from a single colony were incubated overnight in liquid Lysogeny Broth (LB) at 37°C, which were used to inoculate fresh media as described below.

### Determination of final growth

Overnight pre-cultures were centrifuged at 10000 rpm for 5 min, then washed and resuspended in 5 ml fresh LB. Growth values were taken for each isolate (OD_600_) and used to calculate the inoculation volume for a starting OD_600_ of 0.05 in 50 ml LB. Cultures were then incubated with shaking at 37°C for 18 h. Growth was measured by OD_600_. Cultures were then centrifuged at 10000 rpm for 5 min and 40 ml of supernatant was filter sterilised using 0.2 μM filters and used for phenotypic analysis.

### LasA protease assay

LasA protease activity was determined by assessing the ability of *P. aeruginosa* culture supernatants to lyse boiled *Staphylococcus aureus* cells^51^. Overnight cultures of *S. aureus* (RN6390B) were boiled for 10 min and then centrifuged at 4000 rpm for 10 min. The resulting pellet was re-suspended in 0.02 M Tris-HCl (pH 8.5) to an optical density of 0.8 at 600 nm. 190 μL of *S. aureus* suspension was added to 8 replicate wells of a 96 well plate (Greiner). 10 μL of filter sterilised *P. aeruginosa* supernatant was then added to each well. Optical density at 600 nm (A_600_) was measured at 10 min intervals for 60 min using a microplate reader (Tecan Infinite® 200). LasA activity was determined as the percentage of the initial A_600_ value remaining after 60 min.

### LasB protease assay

The elastolytic (LasB) activity of *P. aeruginosa* supernatants was determined using the elastin Congo red (ECR, Sigma) assay^52^. A 100 μl aliquot of filter-sterilized bacterial supernatant was added to 900 μl ECR buffer (100 mM Tris, 1 mM CaCl_2_, pH 7.5) containing 20 mg ECR and incubated with shaking at 37°C for 3 h at 200 rpm. Insoluble ECR was removed by centrifugation and the absorption of the supernatant measured at 495 nm. Buffer containing ECR with and without PAO1 supernatant were used as positive and negative controls respectively. The production of LasB/cell is expressed as the OD_495_ divided by the final growth (OD_600_) previously recorded after 18 h incubation.

### Pyocyanin extraction assay

Pyocyanin was extracted and quantified using a previously described assay^53^. 3 ml of chloroform was added to 5 ml of sterile supernatant and vortexed for 2 min. This was then centrifuged at 10000 rpm for 5 min. The bottom chloroform layer was removed and transferred into a new 50 ml falcon tube to which 2 ml of HCl (0.2 M) was added, mixed and centrifuged. The top 1 ml layer was transferred to a cuvette and the optical density read at 520 nm. Pyocyanin production for each clinical isolate was measured against PAO1.

### QS signal molecule extraction

The method used was based on a previously described assay for the extraction of QS signal molecules^54^. A single colony of each isolate and PAO1 was used to inoculate 10 ml LB and incubated at 37°C at 200 rpm for 18 h. After incubation, the OD_600_ was measured for each sample, centrifuged at 13000 rpm for 5 min and 5 ml of supernatant was filter sterilised. 6 ml of acidified ethyl acetate (0.1% glacial acetic acid) was added to the supernatant, vortexed thoroughly and centrifuged at 10000 rpm for 5 min. 5 ml from the separated top layer was transferred into a glass vial. This was repeated a further two times, and samples pooled into the glass vial after each step. Samples were then dried using a rotary evaporator. 1.5 ml of methanol was added to dried fractions and transferred to a 1.5 ml tube. The sample was dried again under nitrogen gas and re-suspended in 250 μl methanol and stored at −80°C until LCMS analysis. LCMS analysis was performed as previously described^54^. The method was performed in triplicate for all 44 isolates, PAO1 and appropriate media controls.

### Determination of antibiotic susceptibilities using the BSAC method

Bacterial suspensions were made for each isolate by inoculating 1 ml of sterile distilled water (SDW) with a single colony in a 1.5 ml sterile tube to a 0.5 McFarland standard and vortexed gently. Using a sterile cotton swab, each suspension was streaked onto Isosensitest agar (VWR laboratories) twice to create a bacterial lawn. Antibiotic disks were then placed onto the agar using a standardised applicator (Oxoid) (Amikacin 30 μg, Ceftazadime 30 μg, Ciprofloxacin 1 μg, Gentamicin 10 μg, Meropenem 10 μg, Pipericillin/Tazobactam (Tazocin®) 85 μg, Aztreonam 30 μg, Colistin 25 μg, Tobramycin 10 μg and Chloramphenicol 10 μg (Oxoid)). Plates were then inverted and incubated at 37°C for 24 h. The diameter of the zones of inhibition (mm) were measured and compared to pre-determined breakpoints provided by BSAC, with each isolate recorded as either sensitive or resistant to each of the chosen antimicrobials. This experiment was performed in triplicate. All 44 isolates were then mixed as a population in a 1 ml suspension with SDW. Individual colonies per isolate were sampled at random from LB agar streaked with glycerol stocks of the chosen isolate. The population was then diluted to a 0.5 McFarland standard, and the experiment repeated as previously described 8 times. The Nottingham University NHS Trust *P. aeruginosa* control strain (NCTC) was used alongside PAO1 as a control throughout.

### Antibiotic resistance analysis

The mm diameter measurements of clearance zones were first squared so that values were proportional to the area of the clearance zone. To obtain a score of resistance the squared clearance zones were mean standardised within each antibiotic and the negative mean standardised values were taken to be resistance. In this way we generated antibiotic sensitivity scores that could be compared across isolates and across antibiotics. Pairwise correlations were performed using Spearmans rank correlation revealing 12 significantly positively correlated sensitivity profiles (9 remained significant after correction for multiple testing using false discovery rate, as described above). Multi-drug resistance was calculated as the mean resistance score across all antibiotics.

### Genome sequencing and analysis

Genomic DNA was prepared from 14 h cultures of isolates SED1–22. Multiplexed, 150 bp Paired-end sequencing was performed on the Illumina HiSeq2000 platform to an average of 50× coverage. De novo alignments were performed using Velvet and the assemblies optimised using the PAGIT suite of programmes^55^. Assembled genomes were annotated using Prokka. Comparative genomics were performed by pairwise Blast analysis using BRIG^56^ and by Progressive Mauve genome alignments^57^. A pan-genome of the population was created using LS-BSR^58^ and plotted using ggplot2 in R. Breakpoint analysis using paired end reads was performed to detect inversions and rearrangements using BreakDancer^59^, and the resulting alignments visualised with EasyFig^60^. Core genome alignments were performed using Mugsy as previously described^61^. SNP typing was performed using SMALT and Samtools against the LESB58 reference genome, with SNPs in IS, transposons and Phages removed. High fidelity SNPs were then called using a cut off of minimum allele frequency of 0.75, minimum quality score 30, and minimum depth of 8 as described previously^62^. Maximum likelihood phylogenies were created with RaxML implementing the GTRGamma substitution model, and visualised using Figtree. Pairwise SNP distance matrices were created using Mega and visualised by ggplot2 in R. Positive selection on homoplasic SNPs was determined by aligning genes containing SNPs in Mega, and testing for selection using the codon-based Z test for selection. All raw sequence data has been deposited to the European Nucleotide Archive (Accession number: PRJEB5764), and a full set of mapping and assembly statistics are supplied in Supplementary Table S3.

### Statistical association of recombination fragments with phenotypic changes

To account for the linkage of SNPs and to avoid overly conservative results due to simplistic multiple testing correction such as the Bonferroni method, we used the standard approach of permutation association tests, where the isolates are retained while the phenotypic labels of the individuals are randomly permuted^63^. The test procedure for each phenotype was based on 10,000 permutations and the standard ANOVA test statistic where the null hypothesis of no association is compared to a general alternative^63^ using the functions available in the Statistics Toolbox of MATLAB software (v R2012a). As noted in ref. 23, permutation tests are generally considered the gold standard in multiple testing adjustment in genetic association studies, in order to sacrifice as little power as possible, in contrast to for example Bonferroni type correction. SNPs with the permutation-based p-value < 0.05 were defined as significantly associated with the phenotype under consideration. The entire raw data set of p values for each SNP is presented in Supplementary Table S2.

### Recombination analysis

The BratNextGen software^64^ was used to detect recombination events in the 22 genomes, similar to several recent discoveries made using this method^61^,^65^. The whole-genome alignment produced with Mugsy was used as input to BratNextGen with the default settings similar to ref. 64 with 20 iterations of the estimation algorithm. The convergence was assessed to be sufficient since changes in the hidden Markov model parameters were negligible over approximately the last 50% of the iterations. Significance of each recombining region was determined as in ref. 64 using a permutation test with 100 permutations executed in parallel on a cluster computer (threshold of 5% was used to determine significance for each recombination).

### Principal component analysis

Principal component analysis (PCA) was conducted on QS signal and phenotype data using R 2.14.0 (‘R' development core team, 2011) and the *FactoMineR* package. A single PCA on combined signals + virulence factor data was also performed, but this explained less variation than either of the two separate analyses; we therefore present the separate analyses as the more parsimonious approach. Pairwise Spearman's rank correlations were corrected for multiple comparisons using a false discovery rate method^66^.

## Author Contributions

Conceived and designed the experiments: S.P.D., S.E.D. and A.M. Performed the experiments: S.E.D. Analysed the data: S.P.D., S.E.D., A.M. and F.H. Contributed reagents/materials/strains/analysis tools: J.C., H.L.B., A.M., K.P., S.A.C., S.H. and A.F. Wrote the paper: S.P.D., S.E.D., A.M. and F.H.

## Supplementary Material

Supplementary Information SI Figures

Supplementary Information Table S1

Supplementary Information Table S2

Supplementary Information Table S3

## Figures and Tables

**Table 1:** Homoplasic SNPs involved in adaptive evolution

SNP position	CDS	Mutation	No. Haplotypes with mutation	dN/dS
468009	*mexB*	Stop codon	2	10.658
1211690	PLES_11151 – Carboxyl esterase	Val to Ala	2	9.99
1469230	PLES_13621 – Hypothetical protein	synonymous	5	1.12
1469235		synonymous	5	
1469256		synonymous	9	
1469277		synonymous	13	
1469280		synonymous	12	
1469290		Pro to Ser	16	
1469298		synonymous	15	
1469310		synonymous	17	
1469421		synonymous	21	
1469485	PLES_13631 – hypothetical protein	synonymous	19	0.008
1469498		Leu to Iso	21	
1469581		synonymous	18	
1469584		synonymous	18	
1469593		synonymous	11	
1658081	PLES_15261 – NADH quinone reductase	Glu to Ala	3	1.023
1676171	PLES_15441 – mechanosensitive ion channel	Val to Ala	2	1.063
1986123	PLES_18391 – ATP binding permease	Synonymous	2	0.001
2690373	tRNA-Ser	NA	7	
2690375		NA	5	
3020931	*mexT*	Val to Ala	2	1.032
3048652	PLES_28331 – putative haemoylsin secretion/activation protein	synonymous	19	0.009
3048751		synonymous	17	
3058594	PLES28341 – hypothetical protein	Gly to Ala	9	11.478
3058611		Ala to Thr	5	
3090543	*glyA2*	Leu to Iso	16	6.06
3090547		Glu to Arg	11	
3090548		Glu to His	8	
3090609		Iso to Leu	4	
3378695	*psiK*	Synonymous	2	0.001
3378321	*pslJ*	synonymous	7	0.002
3478510	PLES_31551 - hypothetical	Leu to Phe	2	1.098
3502354	PLES_31741 – trehalose synthase	Leu to Val	2	1.049
3973880	Intergenic PLES_35761 - *cysB*	NA	5	
4015334	*pcrV*	Asp to Asn	2	1.104
4026225	*pscT*	Asn to Lys	4	1.088
4539068	PLES_41121 – NAD deacetylase	Val to Ala	4	1.099
5133783	Intergenic PLES_46606 - *sbcD*	NA	7	
5172963	*pctC*	Leu to Pro	5	1.008
6430173	*glyA1*	Glu to Arg	16	0.305
6430175		synonymous	19	
6557923	PLES_59241 – hypothetical protein	Ala to Gly	14	3.227

## Notes

**Ethics Statement.** Spontaneous sputum samples were obtained from a 19 year old female patient with CF, chronically infected with *P. aeruginosa* for 3 years, participating in a concurrent observational study (MRec: 09/H0407/11) and written informed consent was obtained. At the time of sampling, the patient was clinical stable and was prescribed maintenance oral and nebulised therapies only. The board which approved the study was the North Nottinghamshire Ethics Committee. The methods were carried out in accordance with the approved guidelines for this journal.
