# Supplementary material for: Recombination is a key driver of genomic and phenotypic diversity in a Pseudomonas aeruginosa population during cystic fibrosis infection
Source: Sci Rep. 2015 Jan 12;5:7649. doi: 10.1038/srep07649 (PMC4289893; doi:10.1038/srep07649)
Supplement: Supplementary Information — SI Figures [file srep07649-s1.pdf]

Recombination is a key driver of genomic and phenotypic diversity in a *Pseudomonas aeruginosa* population during cystic fibrosis infection

Sophie E. Darch, Alan McNally, Freya Harrison, Jukka Corander, Helen L. Barr, Konrad Paszkiewicz, Stephen Holden, Andrew Fogarty, Shanika A. Crusz, and Stephen P. Diggle

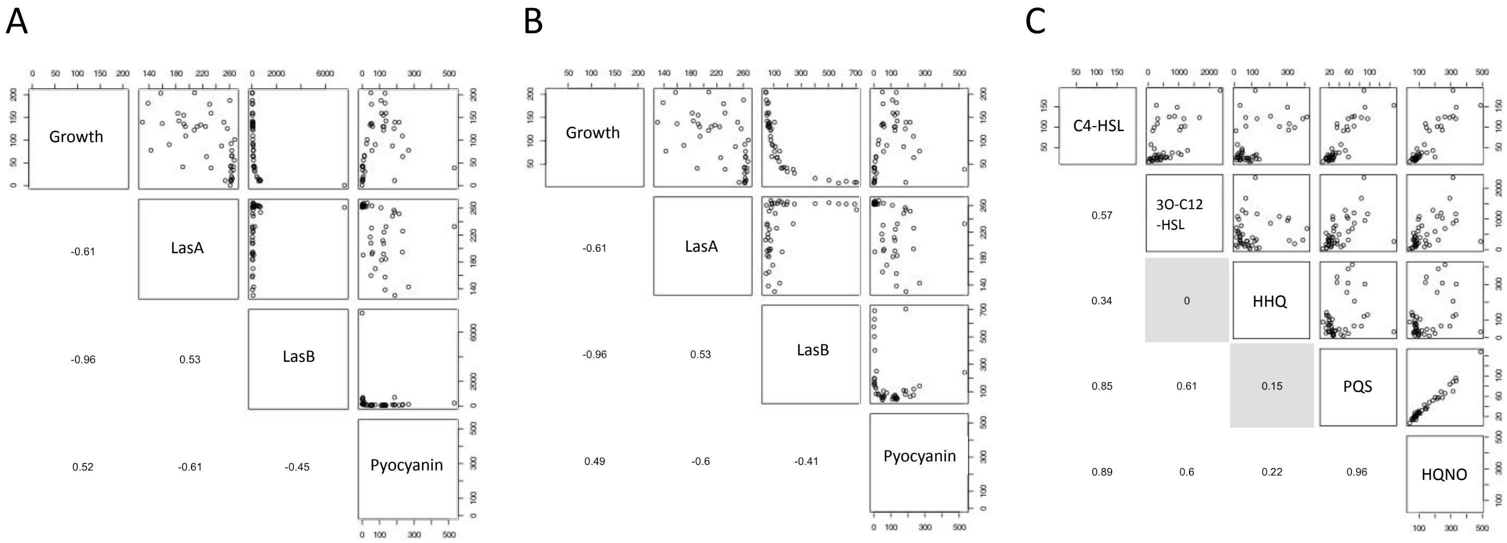

Figure S1. Pairwise correlations between phenotypic traits. Pairwise scatter plots for (A) growth and virulence-related phenotypes; (B) growth and virulence-related phenotypes excluding one outlier (SED23) and (C) QS signal molecules. Numbers given are Spearman's rank correlation coefficients; cells with a grey background denote correlations that did not remain significant ($p > 0.05$) after correction for multiple comparisons.

	LESB58	SED2	SED10	SED20	SED21	SED19	SED16	SED12	SED4	SED18	SED7	SED15	SED3	SED9	SED14	SED22	SED11	SED1	SED13	SED6	SED17	SED5
SED2	36																					
SED10	39	13																				
SED20	41	9	8																			
SED21	42	14	11	17																		
SED19	43	13	8	6	17																	
SED16	44	16	17	21	8	21																
SED12	45	15	10	8	15	6	17															
SED4	47	17	16	18	15	16	15	14														
SED18	47	17	8	14	11	12	15	12	12													
SED7	49	13	10	12	13	10	15	10	14	8												
SED15	50	26	23	27	16	25	16	25	21	19	19											
SED3	50	20	11	13	16	11	20	9	19	9	11	26										
SED9	50	20	11	13	14	13	16	11	13	9	11	24	12									
SED14	52	22	15	17	16	15	18	13	19	13	11	24	10	14								
SED22	52	16	13	13	16	11	18	11	15	9	5	20	8	12	8							
SED11	53	21	22	22	15	22	15	20	22	18	14	17	21	17	15	13						
SED1	54	20	15	19	12	19	14	17	15	13	9	14	18	14	16	14	13					
SED13	54	24	19	19	24	17	24	15	17	17	17	30	14	16	16	12	23	22				
SED6	58	26	23	23	22	21	22	21	19	15	13	24	18	18	14	12	17	16	20			
SED17	59	27	20	22	19	20	21	20	26	16	14	25	13	17	9	11	18	19	23	15		
SED5	59	25	20	20	27	16	31	18	24	20	18	35	15	21	21	15	28	25	17	27	24	
SED8	98	64	61	59	60	57	60	57	59	57	51	58	52	56	48	48	53	48	48	46	43	47

Figure S2. Heatmap visualisation of pairwise SNP distance matrix of the isolates. SNPs were determined by mapping against the reference LESB58 genome sequence.

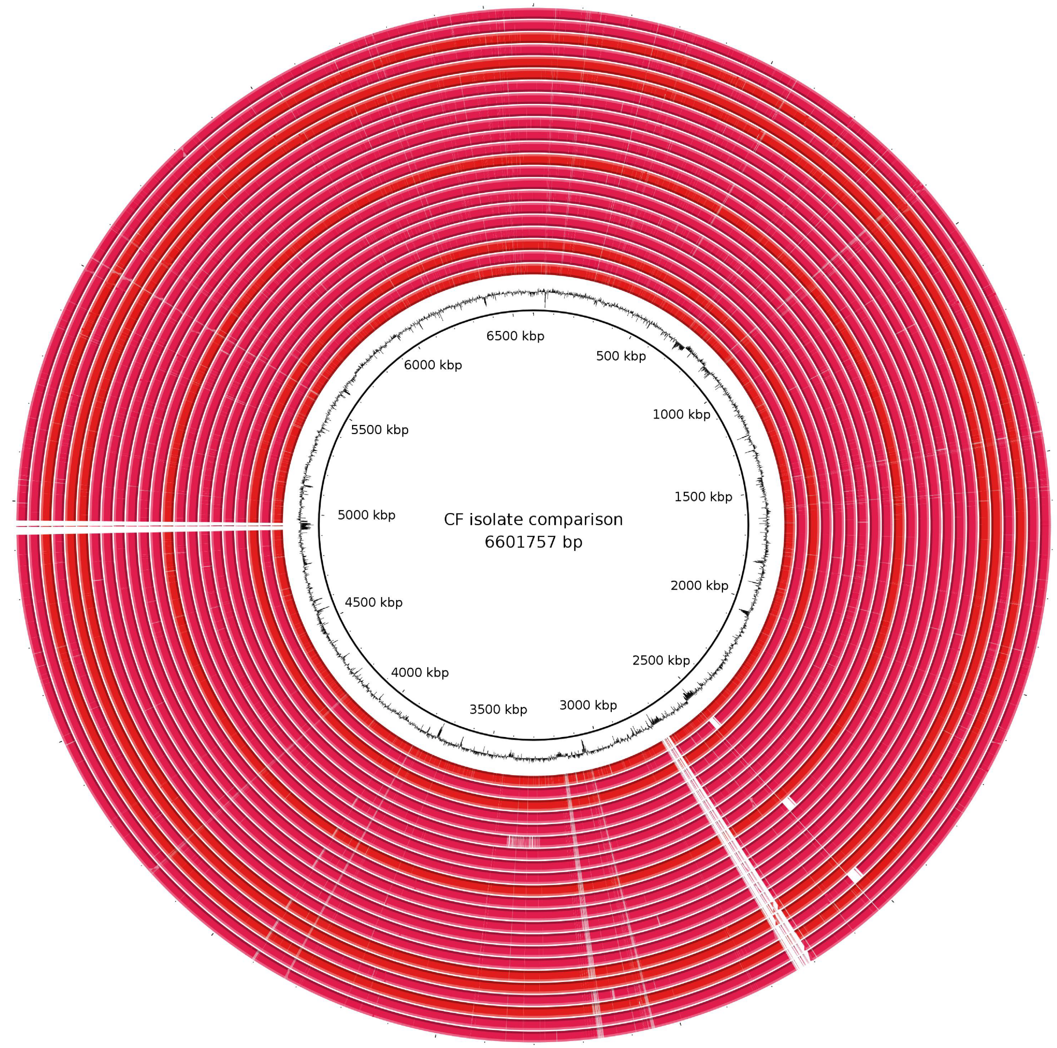

Figure S3. Whole genome comparisons of the assembled genomes of 22 isolates and the reference genome LESB58. Individual circles represent a single isolate, with isolate SED1 the innermost and SED22 the outermost circle. Assemblies indicate identical genomes to LESB58 apart from the deletion of a small prophage in 3 isolates as represented by gaps identified in isolates 2, 11 and 19. No major deletion events are thought to be responsible for the phenotypic diversity displayed in this population.

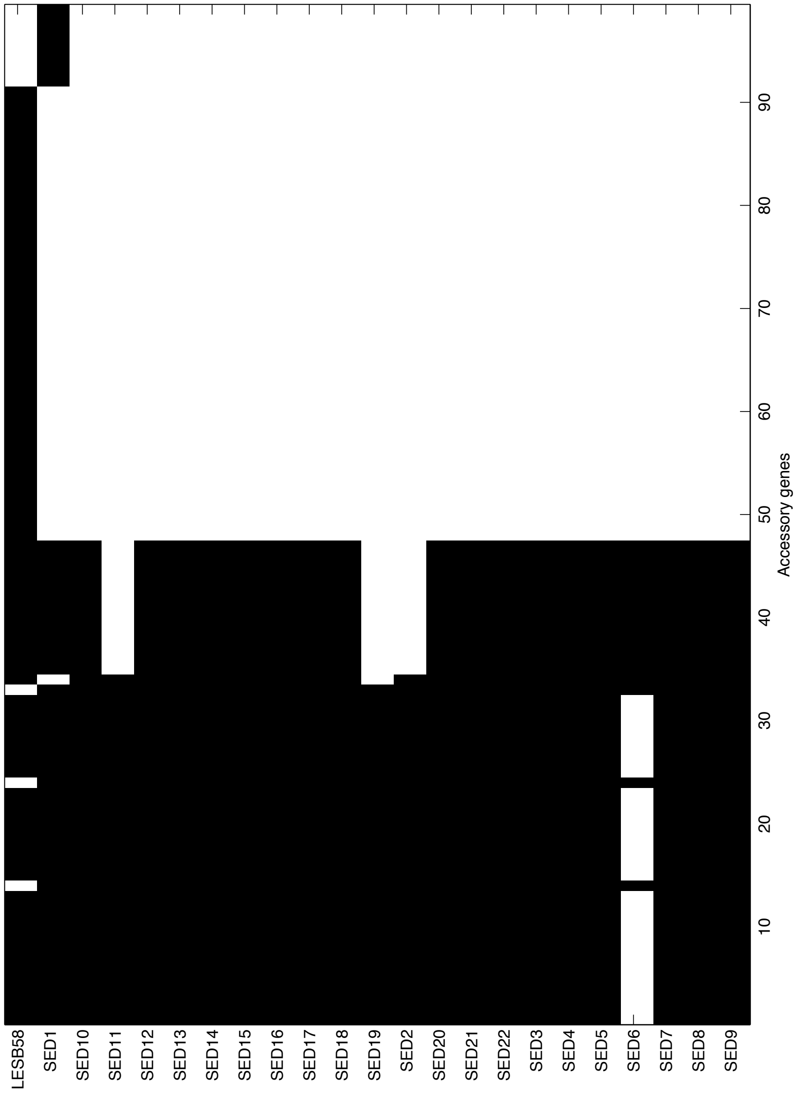

Figure S4. Heatmap showing the distribution of all accessory genes present across the entire genome data set as determined by construction of a pan-genome in LS-BSR. Black indicates presence of a region at 100% identity, whilst white shows absence of a region as defined by the absence of any region showing more than 40% nucleotide sequence identity.

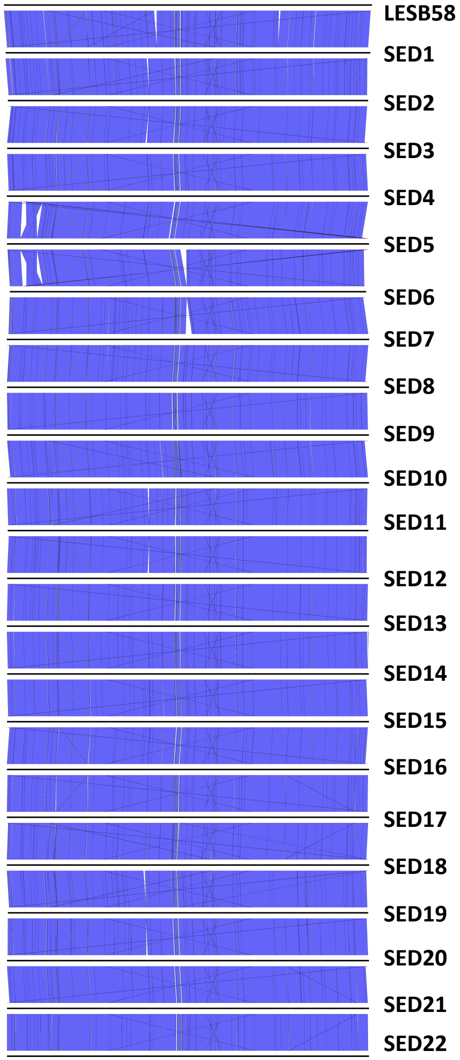

Figure S5. Visualisation of the alignment of individual contigs in each genome rearranged by break point analysis of Paired-end reads.

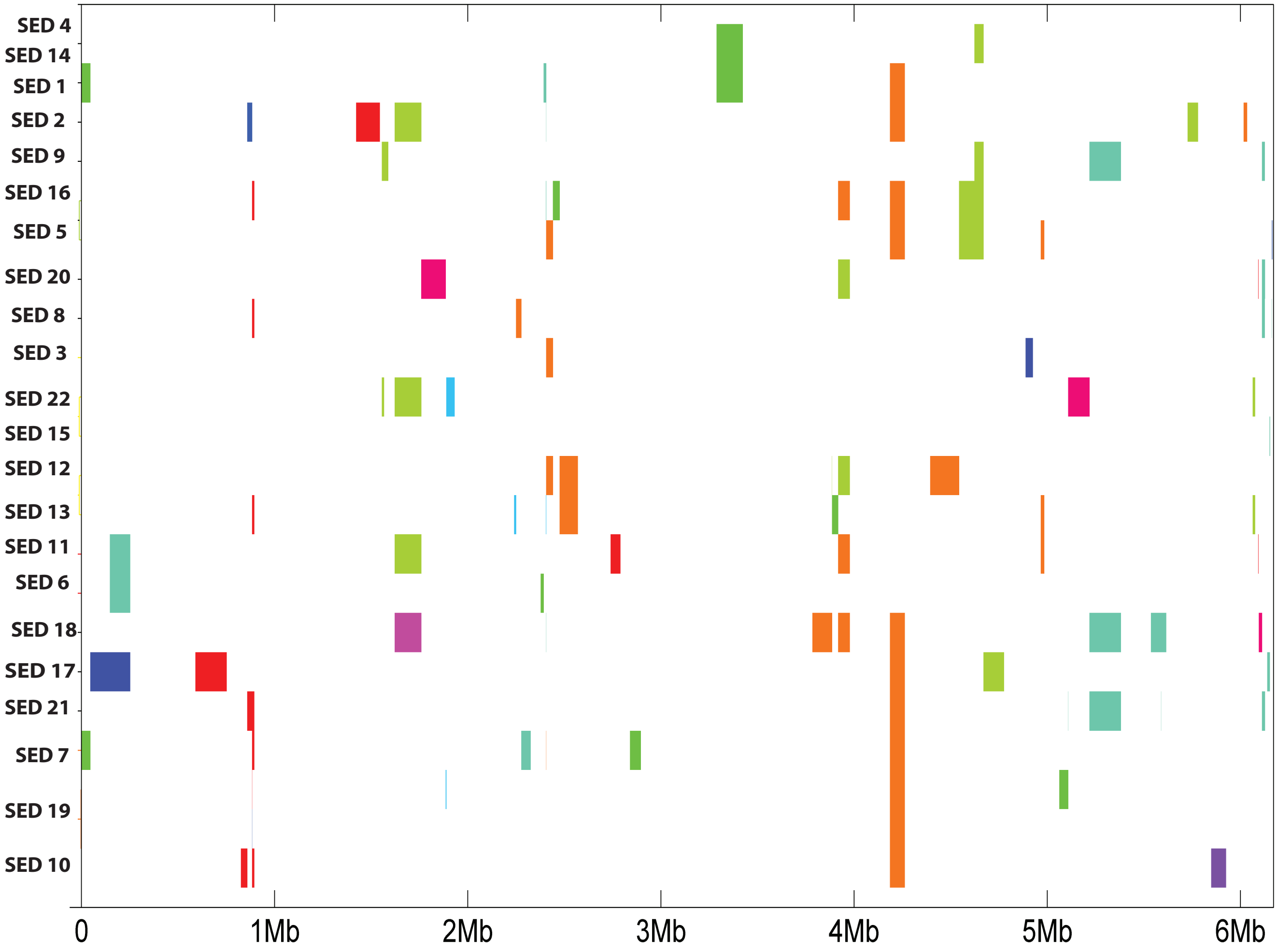

Figure S6. Graphical representation of recombination across the 22 isolate genome sequences as determined by BRATNextGen analysis. Labels to the left indicate each isolate. Each isolate in the analysis is a dash on the y axis of the diagram. The x axis is marked by base pair position relative to the core genome pseudomolecule formed from the whole genome alignment. Bars in the diagram represent regions of recombination detected within the core genome of each strain, with the color coding of the bars allocated in an arbitrary manner.

A

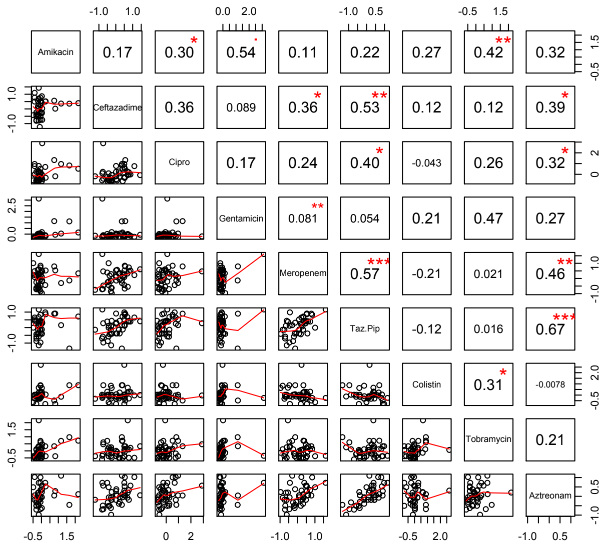

B

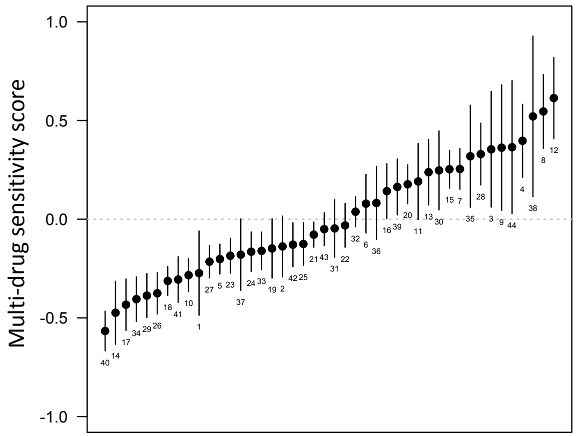

Figure S7. The antibiotic susceptibility of isolates to combinations of CF therapeutics. (a) Pairwise correlations reveal significant positive correlations across several different antibiotics indicating the tendency for multiple drug resistance in some clones. Plotted in the lower triangle are the squared diameter clearance zones (mm) normalised within antibiotic. The values in the upper triangle are spearman rank correlation coefficients and the red stars indicate a p value < 0.05 on the correlation. (b) Multi-drug sensitivity of isolates. Individual isolates vary to the extent that they can resist the effects of multiple antibiotics with some resistant to many antibiotics and others sensitive to many antibiotics. Plotted are the mean sensitivity scores for 9 antibiotics. The vertical lines represent the standard error of the mean and the numbers represent the individual isolates (SED 1-44).

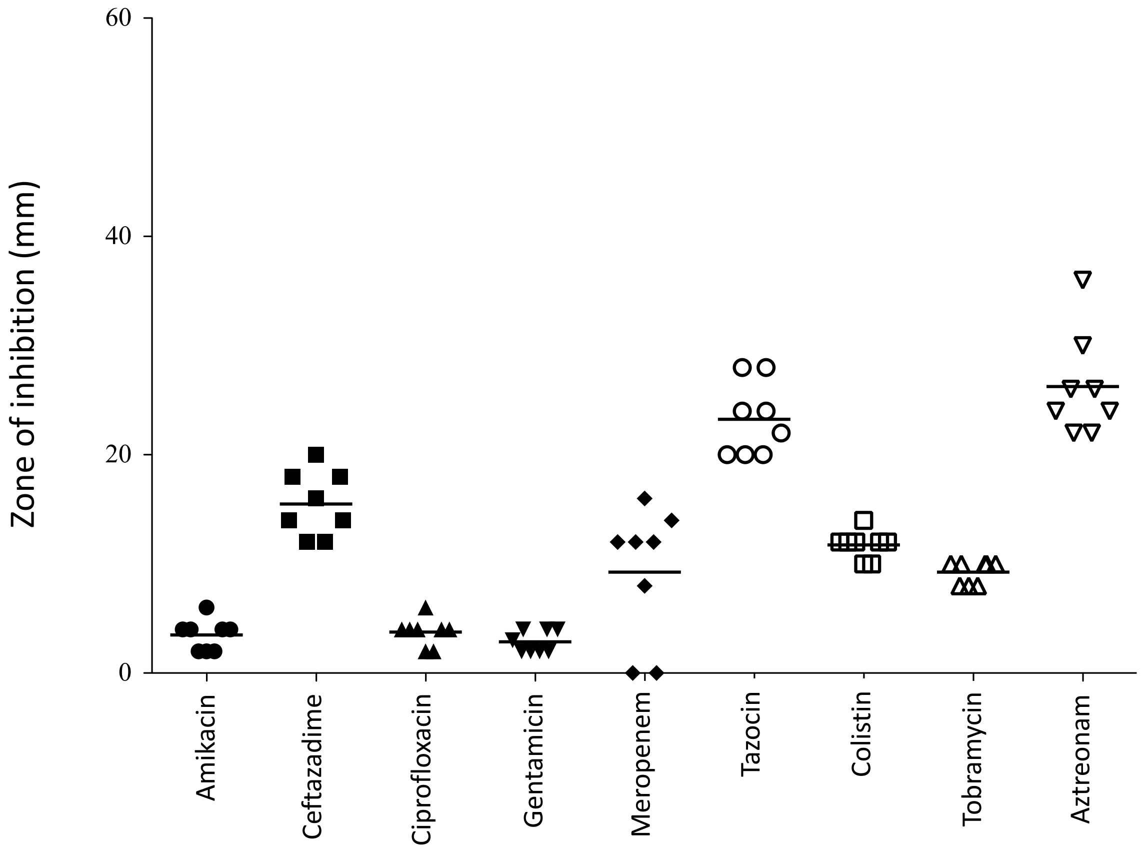

Figure S8. The antibiotic susceptibility of mixed isolates to common CF therapeutics. The recorded zones of inhibition for 44 mixed isolates are shown after replicate antibiotic susceptibility testing to individual isolates was performed. Each data point represents 1 biological replicate of 8 replicates for mixed isolates. The horizontal bars represent the mean value of the zone of inhibition for 8 biological replicates.
